# Supplementary figures and images for: 10-year survival outcome after clinically suspected acute myocarditis in adults: A nationwide study in the pre-COVID-19 era
Source: PLoS One. 2023 Jan 31;18(1):e0281296. doi: 10.1371/journal.pone.0281296 (PMC9888677; doi:10.1371/journal.pone.0281296)

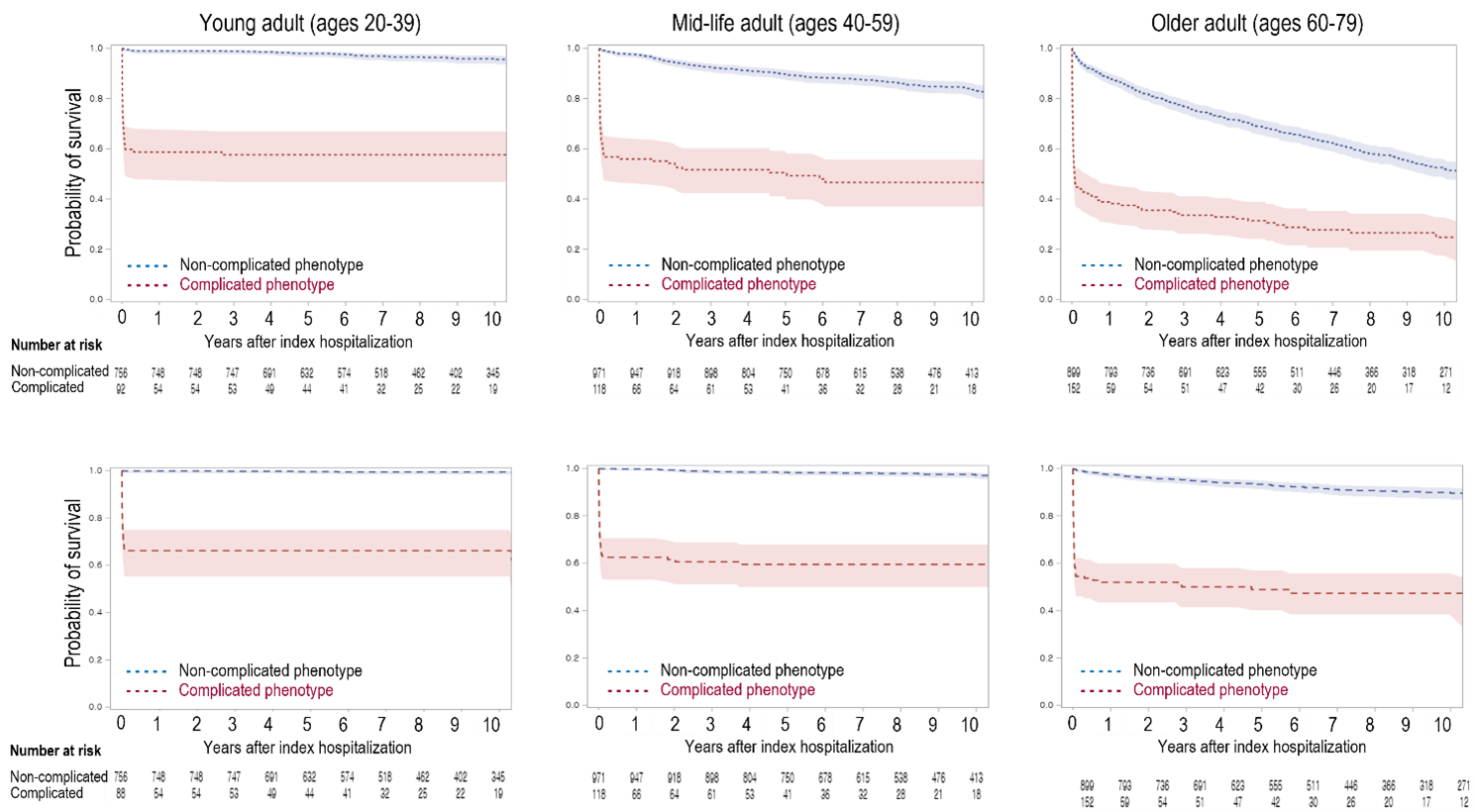

Supplement: S1 Fig — All-cause (upper panel) and cardiovascular (lower panel) mortality according to differential clinical severity. (TIF) [file pone.0281296.s001.tif]

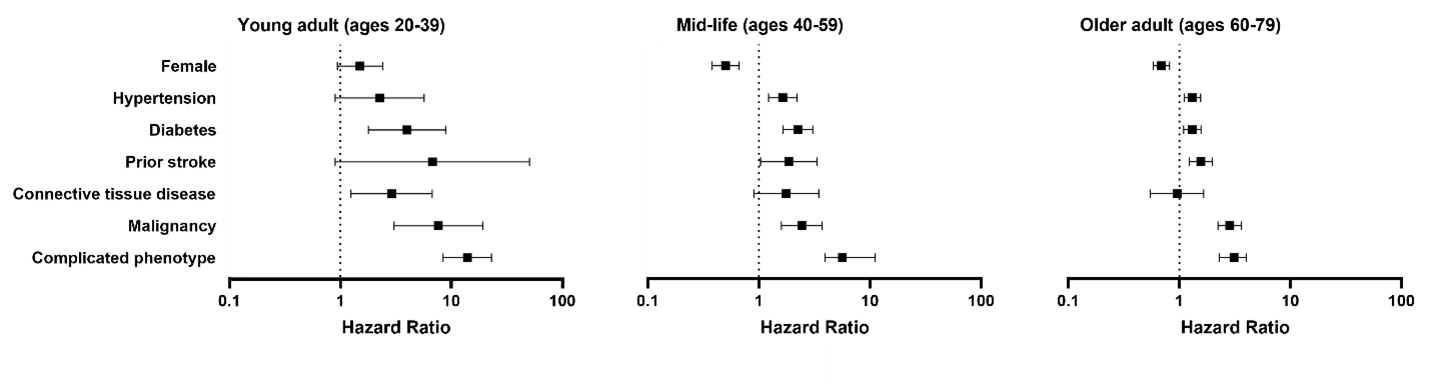

Supplement: S2 Fig — (TIF) [file pone.0281296.s002.tif]

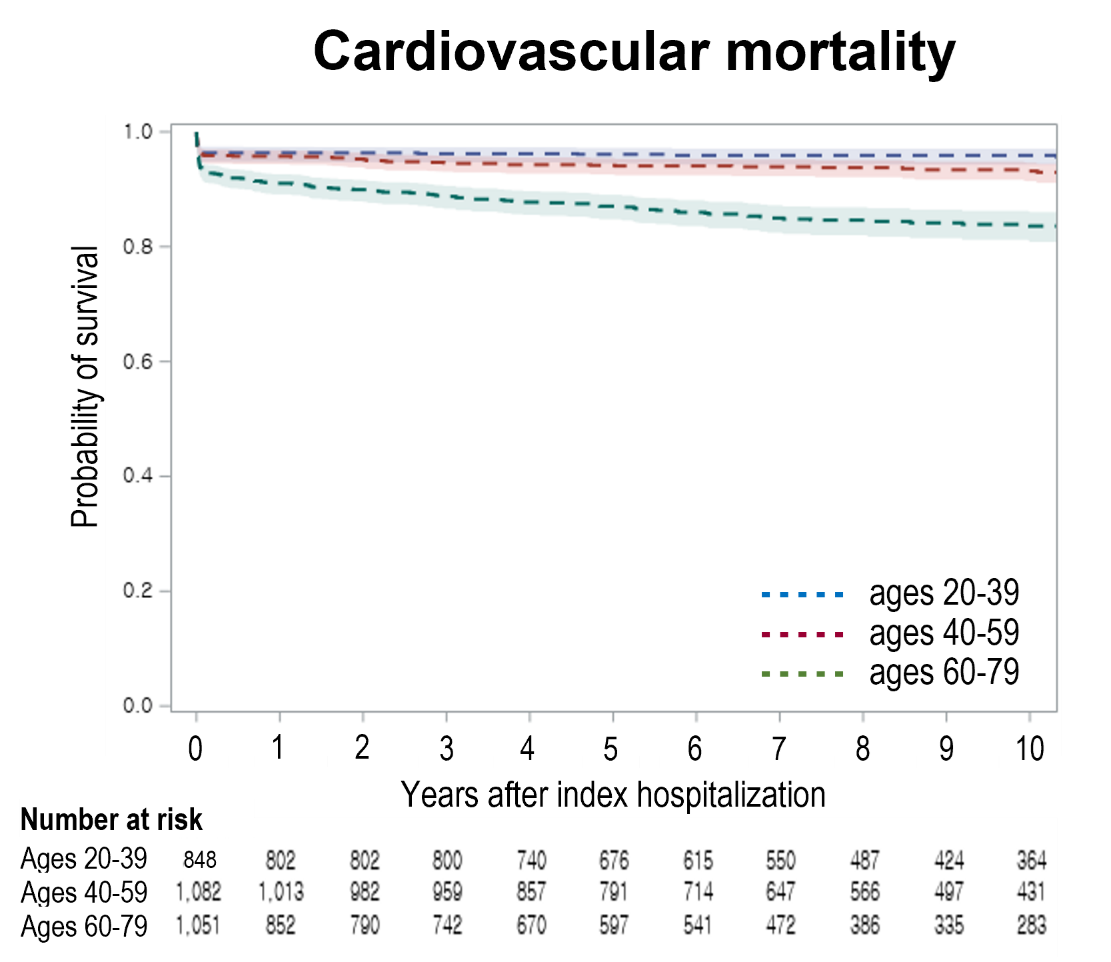

Supplement: S3 Fig — (TIF) [file pone.0281296.s003.tif]
