# Supplementary material for: 10-year survival outcome after clinically suspected acute myocarditis in adults: A nationwide study in the pre-COVID-19 era
Source: PLoS One. 2023 Jan 31;18(1):e0281296. doi: 10.1371/journal.pone.0281296 (PMC9888677; doi:10.1371/journal.pone.0281296)
Supplement: S1 Table — (DOCX) [file pone.0281296.s004.docx]

S1 Table. The annual incidence of suspected acute myocarditis in adults

| Year | Number of cases per 100,000 |
| --- | --- |
| 2006 | 0.569 |
| 2007 | 0.886 |
| 2008 | 0.998 |
| 2009 | 0.689 |
| 2010 | 0.489 |
| 2011 | 0.357 |
| 2012 | 0.576 |
| 2013 | 0.467 |
| 2014 | 0.564 |
| 2015 | 0.501 |
| 2016 | 0.432 |
| 2017 | 0.437 |
| 2018 | 0.491 |
| (Average) | (0.491) |

Incidences were calculated based on the 2009 national resident population of 40,084,082.
