# Supplementary material for: 10-year survival outcome after clinically suspected acute myocarditis in adults: A nationwide study in the pre-COVID-19 era
Source: PLoS One. 2023 Jan 31;18(1):e0281296. doi: 10.1371/journal.pone.0281296 (PMC9888677; doi:10.1371/journal.pone.0281296)
Supplement: Attachment — Submitted filename: 0_response to reviewer.docx [file pone.0281296.s005.docx]

Dear editor and reviewer,

I am so pleased to have the opportunity to revise my manuscript.

I would like to deeply appreciate the editor and reviewer for the time to read our manuscript and for your valuable comments.

First of all, I agree with you that our data was not exactly about acute myocarditis.

This approximated the suspected cases of acute myocarditis; however, it is currently the best available nationwide estimate despite its indirectivity.

Our national database had a fundamental limitation that makes it difficult for us to describe the diagnostic methods applied to our study population according to the current standard guideline.

In our national medical system, governmental medical insurance is applied to the entire residents, but unfortunately, some things are not covered by insurance, such as cardiac MRI for the diagnosis of suspected cases of acute myocarditis. Expensive non-covered items, including cardiac MRI, are required for the personal payment of individual patients. Such practices are performed exceptionally not only because of the costs but also because they might be unintentionally mistaken for over-treatment. Such situations have significantly improved since the pandemic, but there are still no official statistics on the number of unreported cardiac MRIs performed in patients with suspected acute myocarditis at least prior to 2018.

Therefore, in the 2006-2018 period included in our study, at least officially, many acute myocarditis diagnoses were made in a way that excluded alternative diagnoses step by step. To exclude ischemic heart disease, for example, invasive coronary angiography was performed. The diagnosis of acute myocarditis, the starting point of our study, was officially reported by each attending physician to the national data in the middle of such a situation.

We applied an operational definition using inclusion and exclusion criteria.

We excluded potential alternative diagnoses that could be mistaken for acute myocarditis as much as possible, which was made by a detailed reviewing all the diagnostic and procedural ICD codes within 3 months before and after the index hospitalization:

(1) A wide range of acute and chronic ischemic, myocardial, valvular, arrhythmic, and pericardial diseases, genetic and secondary cardiomyopathies, congenital heart diseases, secondary myocardial involvement of endocrine diseases, and iatrogenic cardiac injuries were directly excluded.
(2) Cases with procedural codes of PCI and heart surgeries were excluded.
(3) Cases with a hospital stay of fewer than 5 days were excluded.

What kind of conditions other than acute myocarditis can be included despite these exclusion criteria?

As a result, our acute and long-term survival rates showed reasonable results compared to the literature on acute myocarditis diagnosed by endomyocardial biopsy or MRI as shown in Figure 3. I thought that this result supported that our method was not deceptive.

So, we modified the manuscript as follows:

(1) The term “acute myocarditis” was replaced with “clinically suspected acute myocarditis” in the title, subheadings, and across the text.

(2) More content on the issue of diagnosing acute myocarditis in the present study was added to the limitation part.

(3) For tables and figures, please refer to the following table for your convenience.

| **The original version** | **Current version** |
| --- | --- |
| **Tables** |  |
| Table 1 | Table 1 (not changed) |
| Previous Table 2 | Modified into Fig 2, Fig 4 |
| Previous Table 3 | Modified into Fig 3 |
| **Figures** |  |
| Fig 1 | Fig 1 (not changed) |
| Previous Fig 2 | Modified into Fig 2, S3 Fig |
| Previous Fig 3 | Modified into Fig 4 |
| Previous Fig 4 was removed |  |
| **Supplemental material** |  |
| Previous S1 Fig | Modified into S1 table |
| Previous S2 Fig | S2 Fig (minor revision in style) |
| Previous S3 Fig | Renumbered into S1 Fig |
| - | S3 Fig (separated from previous Fig 2) |

- The previous Table 2 was deleted, and the contents were separately incorporated into Fig 2 and 4.

- The previous Table 3 was deleted, and the contents were modified into Fig 3.

- The previous Fig 2 was minorly modified to focus on all-cause mortality. The previous content on cardiovascular mortality was separated and moved to supplemental material (S3 Fig).

- The previous Fig 3 was renumbered to Fig 4, and numerical information was added.

- Fig 3 was newly added (modified from the previous table 3)

- The previous Fig 4 was deleted.

(4) METHOD section was supplemented with the operational definition of clinically suspected acute myocarditis as mentioned above. We added our database source to the reference list.

**Additional Editor Comments:**

*1. The authors need to include the details/methodology for diagnosing patients with myocarditis. All Figures and Tables should be presented initially in the Results section not in the Discussion.*

🡪 Very thank you so much for the opportunity for revision and your kind comments.

The lack of detailed information about the endomyocardial biopsies and cardiac MRI was the limitation of our study. I explained above about the background.

To mitigate this issue, we replaced the term acute myocarditis with clinically suspected acute myocarditis and have changed the title and text accordingly. In addition, we modified the limitation part for this issue.

All figures and tables have been moved to the Results section.

I look forward to your generous understanding.

**Reviewer comments**

*1. Is the manuscript technically sound, and do the data support the conclusions?*

Reviewer #1: No

🡪 Thank you for your opinion.

I understand this because of the cardiomyopathy diagnostic problem. This background has been described above.

Because I could not present cardiac MRI data, we replaced the term acute myocarditis with clinically suspected acute myocarditis and have changed the title and text accordingly. In addition, we modified the limitation part for this issue.

*3. Have the authors made all data underlying the findings in their manuscript fully available?*

Reviewer #1: No

🡪 As described in the methods section, NHIS is an official database disclosed to the public. It is available to all researchers for sound purposes according to the application process. However, the raw database can be accessed through PCs installed in a designated place and is not allowed to be taken out in accordance with governmental policies. We noticed this at the time of submission.

If this manuscript is decided to be published, I will be happy to upload the protocol and summary statistics that we currently possess.

*4. Is the manuscript presented in an intelligible fashion and written in standard English?*

*Reviewer #1: No*

🡪 I am sorry for the language barrier, and I would like to thank you for your hard time reading this manuscript.

The manuscript was performed in English editing again by a native speaker with a Ph.D. degree through a commercial company (<https://www.editage.co.kr/>, task ID: ENPJX_1_3).

*5. Review Comments to the Author*

*Reviewer #1: A crucial information that is missing is how the patients were diagnosed with myocarditis. It is important to know, how many patients underwent endomyocardial biopsy and how many CMR to get an idea, how accurate the diagnosis of myocarditis was.*

Thank you so much for your comments. I hope that my excuse would be satisfactory to you.
